# Supplementary material for: Dissociable effects of dopaminergic medications on depression symptom dimensions in Parkinson’s disease
Source: medRxiv. 2023 Jun 30:2023.06.30.23292073. Preprint. [Version 1] doi: 10.1101/2023.06.30.23292073 (PMC10327242; doi:10.1101/2023.06.30.23292073)
Supplement: Supplement 1 [file media-1.pdf]

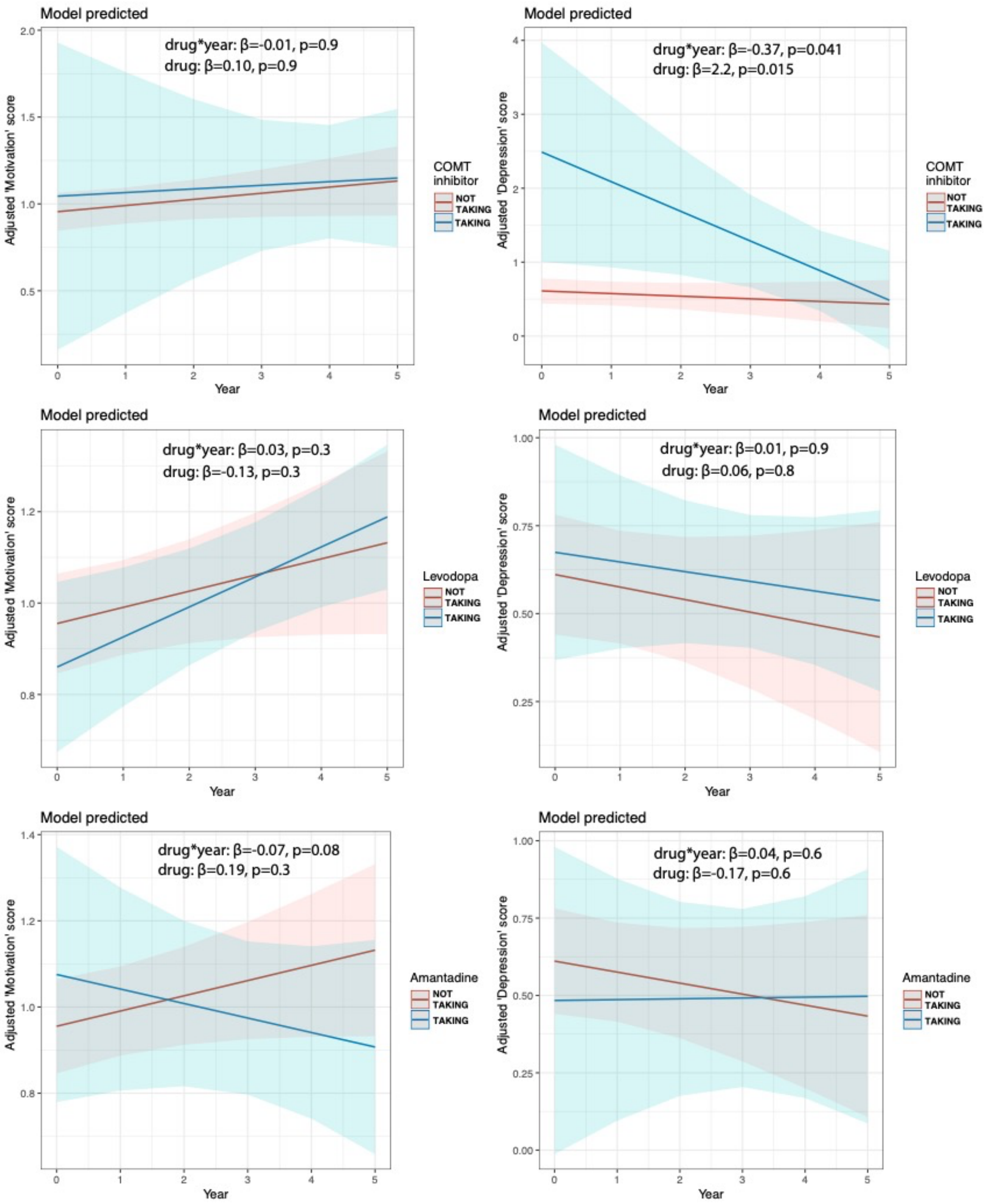

Supplement figure S1. Top row. Adjusted model predicted relationship between COMT inhibitor treatment and 'motivation' factor score and 'depression' factor score over time. **Middle row.** Adjusted mixed effects model of predicted relationship between levodopa treatment and 'motivation' factor score and 'depression' factor score over time. **Bottom row.** Adjusted model predicted relationship between amantadine treatment and 'motivation' factor score and 'depression' factor score over time.
